# Stromal cartilage oligomeric matrix protein as a tumorigenic driver in ovarian cancer via Notch3 signaling and epithelial-to-mesenchymal transition

**DOI:** 10.1186/s12967-024-05083-0

**Published:** 2024-04-13

**Authors:** Gilar Gorji-Bahri, B. Madhu Krishna, Catharina Hagerling, Akira Orimo, Karin Jirström, Konstantinos S. Papadakos, Anna M. Blom

**Affiliations:** 1 Department of Translational Medicine, Lund University, Malmö, Sweden; 2 Department of Experimental Medical Science, Lund University, Lund, Sweden; 3 Department of Pathology and Oncology, Juntendo University, Tokyo, Japan; 4 Department of Clinical Sciences Lund, Lund University, Lund, Sweden; 5 Division of Medical Protein Chemistry, Department of Translational Medicine, Lund University, Malmö, Sweden

**Keywords:** Ovarian cancer, Stroma, COMP, CAF, Epithelial-to-mesenchymal transition

## Abstract

**Background:**

Cartilage oligomeric matrix protein (COMP), an extracellular matrix glycoprotein, is vital in preserving cartilage integrity. Further, its overexpression is associated with the aggressiveness of several types of solid cancers. This study investigated COMP’s role in ovarian cancer, exploring clinicopathological links and mechanistic insights.

**Methods:**

To study the association of COMP expression in cancer cells and stroma with clinicopathological features of ovarian tumor patients, we analyzed an epithelial ovarian tumor cohort by immunohistochemical analysis. Subsequently, to study the functional mechanisms played by COMP, an in vivo xenograft mouse model and several molecular biology techniques such as transwell migration and invasion assay, tumorsphere formation assay, proximity ligation assay, and RT-qPCR array were performed.

**Results:**

Based on immunohistochemical analysis of epithelial ovarian tumor tissues, COMP expression in the stroma, but not in cancer cells, was linked to worse overall survival (OS) of ovarian cancer patients. A xenograft mouse model showed that carcinoma-associated fibroblasts (CAFs) expressing COMP stimulate the growth and metastasis of ovarian tumors through the secretion of COMP. The expression of *COMP* was upregulated in CAFs stimulated with TGF-β. Functionally, secreted COMP by CAFs enhanced the migratory capacity of ovarian cancer cells. Mechanistically, COMP activated the Notch3 receptor by enhancing the Notch3-Jagged1 interaction. The dependency of the COMP effect on Notch was confirmed when the migration and tumorsphere formation of COMP-treated ovarian cancer cells were inhibited upon incubation with Notch inhibitors. Moreover, COMP treatment induced epithelial-to-mesenchymal transition and upregulation of active β-catenin in ovarian cancer cells.

**Conclusion:**

This study suggests that COMP secretion by CAFs drives ovarian cancer progression through the induction of the Notch pathway and epithelial-to-mesenchymal transition.

**Supplementary Information:**

The online version contains supplementary material available at 10.1186/s12967-024-05083-0.

## Background

Ovarian cancer is a significant cause of cancer-related deaths in women worldwide [1]. The most common type is high-grade serous epithelial ovarian cancer, typically diagnosed at late stages with a 5-year cause-specific survival of 26–42% [2, 3]. To improve outcomes, early detection and the discovery of new diagnostic markers as well as mechanisms governing tumorigenesis are essential in combating this life-threatening disease.

Cartilage oligomeric matrix protein (COMP), also known as thrombospondin 5, is a pentameric extracellular matrix glycoprotein. Each monomer comprises four epidermal growth factor domains, eight thrombospondin type 3 domains, and a C-terminal globular domain [4, 5]. COMP is predominantly expressed in cartilage, tendons, and ligaments and is vital for their organization and integrity [6]. *COMP* gene mutations cause skeletal diseases like pseudoachondroplasia and multiple epiphyseal dysplasia [7, 8]. Recently, COMP was found to be highly expressed in several solid cancer types, including colon, colorectal, prostate, liver, and breast cancer [9–16]. COMP overexpression in tumor cells was highly associated with poorer OS and recurrence-free survival of breast and prostate cancer patients [9, 11]. Additionally, serum levels of COMP have been shown to be independent prognostic factors for breast and colon cancer [10, 16]. Studies have provided compelling evidence of COMP’s multifaceted role in cancer biology. COMP protects cancer cells from endoplasmic reticulum stress-mediated apoptosis and induces a metabolic switch, i.e., the Warburg effect [11]. It also interacts with Transgelin, influencing EMT in highly metastatic colorectal cancer [12]. We recently showed that COMP mediates the Notch3-Jagged1 interaction, enhancing breast cancer stem cells associated with AKT, PI3K, and β-catenin signaling pathways [13]. Further, the phosphorylation of ERK and AKT in COMP-treated hepatocellular carcinoma cells resulted in the activation of MEK/ERK and PI3K/AKT pathways and eventually induced the migration ability of cells [15]. Recently, a proteomics analysis showed that COMP was the most highly expressed protein in the stroma of metastatic ovarian tumors, regulated by nicotinamide N-methyltransferase [17]. However, the mechanism of action of COMP and its clinical correlation with ovarian cancer remains unexplored. Moreover, despite understanding the role of COMP in extracellular matrix organization, its influence on the tumor microenvironment (TME) is not fully understood. Therefore, our study aimed to explore the potential links between COMP and clinicopathological characteristics of epithelial ovarian cancer and to unravel the mechanism by which COMP is driving the progression of ovarian cancer.

## Methods

### Cohort

The present study included all incident epithelial ovarian cancer cases from the population-based prospective cohort studies Malmö Preventive Medicine (MPP) (n = 108) [18] and Malmö Diet and Cancer (MDC) (n = 101) [19] until Dec 31st, 2008. A more detailed description of the cohort is provided in previous publications [20–23]. The Lund University Ethics Committee approved the MDCS (Ref. 51/90) and the present study (Ref. 530/2008).

### Immunohistochemistry

Preparation of tissue microarrays and immunohistochemical staining were performed as previously described [24]. The intensity of COMP was evaluated independently by two researchers in a blinded fashion using scores: 0 for negative staining, 1 for low expression, 2 for moderate expression and 3 for high expression. Staining in cancer cells was evaluated separately from the stroma.

To evaluate metastases into the lungs of mice, the first fifteen 5 μm cuts were discarded. The next tissue cut was mounted on Superfrost Plus Adhesion Microscope slides (Epredia). The antigen retrieval was performed with the heat-induced epitope retrieval method using 10 mM citrate buffer (pH = 6). Human metastatic cells were stained with an anti-pan-cytokeratin antibody (Sigma) followed by a Signalstain Boost IHC detection reagent (Cell Signaling Technology). ImmPACT DAB (Vector Laboratories) was then used for the detection of positive cells. The final mounted samples were scanned with the Aperio Scanner system (Leica) at 40X, and the number of positive cells was evaluated by QuPath software [25].

### Xenograft ovarian cancer mouse model

SKOV3 and CAF cells were harvested utilizing Versene solution (Thermofisher, USA), and were resuspended in PBS in a 1:3 ratio of SKOV3 (2 × 10^6^) to CAF2 cells (6 × 10^6^). Cell viability was evaluated using trypan blue, before injection. The transplantation was performed subcutaneously into the flanks of ten-week-old NXG female mice (JanvierLabs, France; n = 10 mice per group). At the endpoint of the experiment, tumor and lung tissues were collected according to the ethical permission. The animal experiment was approved by the local Ethical Committees for Animal Experimentation in Lund, application (4349/2020).

## Cell culture

SKOV3 (ATCC, HTB-77), NIH/3T3 (ATCC, CRL-1658), OAW42 (Sigma, 85073102), and ID8 (Merck, SCC145) cell lines were purchased and cultured in mediums recommended by the manufacturers. Breast cancer-associated fibroblasts (CAF) were generated by Prof. Orimo, Juntendo University, Japan [26]. To prepare stable monoclonal cells, COMP expressing (COMP) and an empty pcDNA3 vector (mock) were transfected to CAF and NIH/3T3 cells (9 × 10^5^) using lipofectamine 3000 transfection reagent (Cat: L300008, Invitrogen, USA). Cells were then treated with 0.7 mg/ml G418. Stable colonies were then picked up and cultured for a month under G418 exposure. ELISA further confirmed the secretion of COMP by COMP-expressing CAFs. All cell lines were maintained at 37 °C in a humidified incubator with 5% CO2 and were checked monthly for mycoplasma contamination using the VenorGeM classic kit (Minerva Biolabs). The passage number of cells in all experiments was below 10.

### Cell proliferation assay

In brief, 3 × 10^4^ cells were seeded in 96-well cell-binding black plates (Cat: 3340, Corning, USA) for 24 h. Cells were then treated with increasing concentrations of COMP for another 24 h. After that, cells were incubated with the detection reagent for one h at 37 °C, and fluorescence intensities were then measured by Cytation 5 Cell Imaging Multimode Reader (BioTek, USA) at 480/535 nm. The percentage of cell survival was calculated and compared to untreated cells as a control.

A coculture assay was performed to evaluate the effect of secreted COMP by COMP-expressing CAFs on the proliferation of SKOV3 cells. In brief, SKOV3 cells were first stained using a CellTrace CFSE cell proliferation kit (Cat: C34554, Invitrogen), according to the manufacturer’s instructions. An equal number (1 × 10^5^) of CFSE-labeled SKOV3 cells and COMP-expressing CAFs or the counterparts mock control CAFs were mixed and seeded in 24-well plates. Unstained SKOV3, COMP-expressing CAFs and the mock control CAFs were used as negative controls to gate out CFSE negative cells. Cells were collected, resuspended in PBS at each time point and analyzed by flow cytometry (Beckman Coulter CytoFLEX). The shift of the CFSE positive peak to the left was evaluated as a measure of the SKOV3 cells proliferation, as CFSE dye was lost with each cell division.

### COMP binding assay

SKOV3 and OAW42 cells (3 × 10^5^) were resuspended in FACS binding buffer (10 mM HEPES, 140 mM NaCl, 5 mM KCl, 1 mM MgCl_2_, 2 mM CaCl_2_, and 0.02% NaN_3_, (pH:7.2)) and incubated with increasing concentrations of recombinant COMP for 1 h. Cells were then incubated with an anti-human COMP primary antibody (homemade) and Alexa-flour 488 conjugated secondary antibody (Invitrogen, USA), respectively, for 1 h at 37 °C. Untreated cells were considered as a control. COMP binding to the cells’ surface was finally evaluated by flow cytometer (Beckman Coulter CytoFLEX, CA, USA), and data were analyzed using the FlowJo software (BD Life Sciences).

### Migration & invasion assay

SKOV3 (4 × 10^4^) and OAW42 (3 × 10^4^) cells were suspended in a serum-free medium with increasing COMP concentrations and then seeded in 24-well plate transwell inserts (PET, 8 μm, Falcon, USA) for a 24-h migration assay or Biocoat Matrigel invasion chambers (Corning, USA) for a 48-h invasion assay. Untreated cells served as a control, and the medium in the lower chamber was supplemented with 10% FBS as a chemoattractant. After steps of washing with PBS, fixing by 3.7% formaldehyde, and staining the cells with a 0.5% crystal violet solution, respectively, the chamber's inner cells were removed using cotton swabs.

In the co-culture assay, mock or COMP-expressing 3T3 or CAF cells (1.5 × 10^5^) were seeded in 24-well plates with 10% FBS and incubated for 3 days. Subsequently, ID8 or ovarian cancer cells (SKOV3 and OAW42) (3 × 10^4^) were seeded in the upper chamber of transwell inserts for 24 h. Staining was performed as described earlier. Images from different areas of each chamber were captured at 4 X or 10 X magnification, using the EVOS XL Core Cell Imaging System (Thermo Scientific, MA, USA) and analyzed with ImageJ software.

### ALDH activity assay

SKOV3 and OAW42 cells were seeded in 6-well plates and treated with 20 μg/ml COMP or PBS as a control for 48 h. Cells were then trypsinized, and 5 × 10^5^ cells per sample were collected to determine the ALDH activity using the ALDEFLUOR assay kit (STEMCELL Technologies, Canada) according to the manufacturer’s instructions. DEAB, a selective inhibitor of ALDH, was used as a control of background fluorescence. Cells were analyzed by flow cytometer (Beckman Coulter CytoFLEX, CA, USA), and data analysis was performed using the FlowJo software (BD Life Sciences).

### Apoptosis assay

Cells (1 × 10^5^) were initially seeded in 6-well plates for 24 h, followed by treatment with COMP (20 μg/ml), BSA (20 μg/ml) (negative control), and cisplatin (positive apoptosis inducer control) either individually or in combination with COMP for 48 h. SKOV3 and OAW42 cells were treated with 10 μM and 20 μM of cisplatin, respectively. Subsequently, the cells were trypsinized and washed with FACS binding buffer (10 mM HEPES, 140 mM NaCl, 5 mM KCl, 1 mM MgCl_2_, 2 mM CaCl_2_, and 0.02% NaN_3_, (pH:7.2)). Incubation with 5 μl Annexin V and 0.4 μl Zombie aqua was carried out for 30 min at room temperature. After washing with FACS binding buffer, the cells were subjected to flow cytometry (Beckman Coulter CytoFLEX, CA, USA), and data was analyzed using the FlowJo software (BD Life Sciences).

### RNA isolation, cDNA synthesis, and RT-qPCR

For recombinant COMP treatment, 80% confluent cells in 6-well plates were treated with recombinant COMP (20 μg and 50 μg/ml) or BSA (50 μg/ml) as a control for 24 h. For cell treatment with TGFβ proteins, 80% confluent cells in 12-well plates were incubated with 10 ng/ml TGFβ1, TGFβ2, TGFβ3, BSA, and the same volume of PBS as a control for 48 h. Then, the medium was refreshed, and cells were incubated for 48 h again. Subsequently, total RNA was isolated from cells using RNeasy Plus Mini Kit (Qiagen, Germany), according to the manufacturer’s instructions. The purity of isolated RNA was checked by measuring the 260/280 absorbance ratio via Nanodrop. The RNA integrity number (RIN) of isolated RNA samples was also checked using an Agilent 2100 Bioanalyzer instrument with an Agilent RNA 6000 Nano kit (Agilent Technologies). Total RNA samples were then quantified and used for cDNA synthesis immediately. Otherwise, they were stored at −80 °C. Total RNA (500 ng) was reverse transcribed to cDNA using SuperScript™ IV First Strand Synthesis System (Cat: 18091050, Thermo Scientific, USA), employing Oligo (dT)_20_, according to the manufacturer’s instructions and stored at −20 °C. RT- qPCR was performed using TaqMan probes (Thermo Scientific, MA, USA) for each gene. *GAPDH* and *HPRT1* were considered internal controls, and relative mRNA expression for each gene was calculated using 2^−ΔΔCt^ or 2^−ΔCt^ as indicated in the figure legend. The array of epithelial to mesenchymal transition genes was performed using PrimePCR Custom Plate (Cat: 10034487, Bio-Rad) according to the manufacturer’s protocol. Results were analyzed using *GAPDH* as an internal control, and were presented in volcano plots.

### Western blotting

Cell lysates were prepared using cold 1X radioimmunoprecipitation assay lysis buffer (RIPA) (10 mM Tris–HCl (pH: 7.2), 150 mM NaCl, 0.1% SDS, 1% Triton X-100, 1% deoxycholate) supplemented with 1% (v/v) Halt Protease and Phosphatase inhibitor Cocktail (100X) (Thermo Scientific, USA). Protein concentrations were quantified using the Pierce BCA Protein Assay Kit (Thermo Scientific, USA). After protein separation on SDS-PAGE gels and transferring to PVDF membranes, blots were incubated with respective primary antibodies, and HRP-conjugated secondary antibodies. The used antibodies are listed in Additional file 1: Table S1. Blots were then visualized under Chemidoc (Bio-Rad, USA) using Immobilon Western Chemiluminescent HRP substrate (Millipore, MA, USA). The bands’ intensities were measured by Image Lab software (Bio-Rad) and normalized to the expression of β-tubulin or GAPDH as a control.

### Proximity ligation assay

In brief, 2 × 10^4^ cells were seeded in a 12-well removable chamber (ibidi, Germany) for 24 h, and then they were treated with 0.25 mg/ml recombinant COMP. After 24 h, cells were fixed with 4% paraformaldehyde, permeabilized with 0.1% triton-X100, blocked with 3% BSA blocking buffer, and then incubated with primary antibodies for 1 h at room temperature, respectively. Respective isotype control antibodies were included as a negative control. Subsequently, incubation with the goat-mouse probes and three steps of enzymatic reactions were performed based on the NaveniFlex GM instructions. Duolink in situ mounting medium with DAPI (Sigma, USA) was used for nuclear staining. At least three images from different areas of each chamber were captured at 63 X magnification under a Confocal microscope (Zeiss LSM800, Germany). Images were analyzed by the ImageJ software to count the spots per cell values.

### Tumorsphere formation assay

Cells (5 × 10^4^) were suspended in the Mammocult medium (STEMCELL Technologies, Canada) supplemented with 4 μg/ml heparin (STEMCELL Technologies, Canada) and 0.48 μg/ml hydrocortisone (STEMCELL Technologies, Canada) to prepare a serum-free single cell suspension. Then, cells were seeded in 6-well Ultra-low binding plates (Corning, USA) with increasing concentrations of COMP and incubated for a week at 37 °C in a humidified incubator with 5% CO_2_. For the NOTCH inhibition studies, cells were incubated with either COMP alone (20 μg/ml) or in combination with NOTCH inhibitors, DAPT (1 μM, Sigma-Aldrich, USA), and anti-Jagged1 antibody (2 μg/ml, R&D systems, USA). A minimum of 10 images per well were captured using the EVOS XL Core Cell Imaging System (Thermo Scientific, MA, USA). The length of at least 10 tumor spheres per well was measured using the ImageJ software.

### Luciferase reporter assay

In brief, 1.4 × 10^5^ SKOV3 cells were seeded in a 24-well plate and incubated overnight to reach 70% confluency on the day of transfection. Cells were then transfected with the following plasmids using lipofectamine 3000 (Invitrogen): the positive control: 2488 ng M50 Super 8 × TOPFlash [27], 2 ng pIS2 [28], and 10 ng pcDNA3-S33Y β-catenin [29]. The negative control: 2498 ng M51 Super 8 × FOPFlash (TOPFlash mutant) [27] and 2 ng pIS2. For the detection of β-catenin activation: 2498 ng M50 Super 8 × TOPFlash, 2 ng pIS2. The next day culture media was replaced, cells were treated with BSA (50 μg/ml), BSA in combination with DAPT (1 μM), COMP (50 μg/ml), and COMP in combination with DAPT (1 μM), and incubated for 24 h. Untreated cells were included as a control. Luciferase activity was detected using a Dual-luciferase reporter assay system (Promega) according to the manufacturer’s instructions. Luminescence was measured via Cytation 5 Cell Imaging Multimode Reader.

Plasmid pIS2 was a gift from David Bartel (Addgene plasmid # 12177), M50 Super 8 × TOPFlash and M51 Super 8xFOPFlash were a gift from Randall Moon (Addgene plasmid, # 12456 and #12457), and pcDNA3-S33Y Beta-catenin was a gift from Eric Fearon (Addgene plasmid # 19286).

### Recombinant COMP purification

In brief, Freestyle 293-F cells (ThermoFisher Scientific, USA) were cultured in FreeStyle 293 Expression Medium (Thermo Scientific, MA USA) supplemented with 1% (v/v) penicillin–streptomycin. Cells were transfected with histidine tagged-COMP pCEP4 plasmid using Freestyle Max transfection reagent (Thermo Scientific, USA). Transfected cells were cultured for 10 days, and the supernatant was collected every 2 days and stored at −20 °C. A Ni–NTA affinity column (Ni–NTA Superflow, Qiagen) loaded in ÄKTAprime plus machine (GE Healthcare) was used for COMP purification. Fractions containing COMP protein were subsequently pooled and dialyzed against 1X PBS (pH = 7.4) and concentrated by 10 kDa ultra centrifugal filter units (Millipore, MA, USA). The purified recombinant COMP protein was finally stored at −80 °C for further experiments.

### Statistical analysis

SPSS software (version 29) was used for survival analyses. GraphPad Prism was used for all other statistical analyses. The data were represented as mean ± standard deviation (SD). To calculate the *p*-value, the student’s T-test, one-way ANOVA, and two-way ANOVA were utilized. *P* value < 0.05 was considered statistically significant.

## Results

### Stromal expression of COMP is associated with adverse clinicopathological factors and a shorter OS

**Fig. 1:**
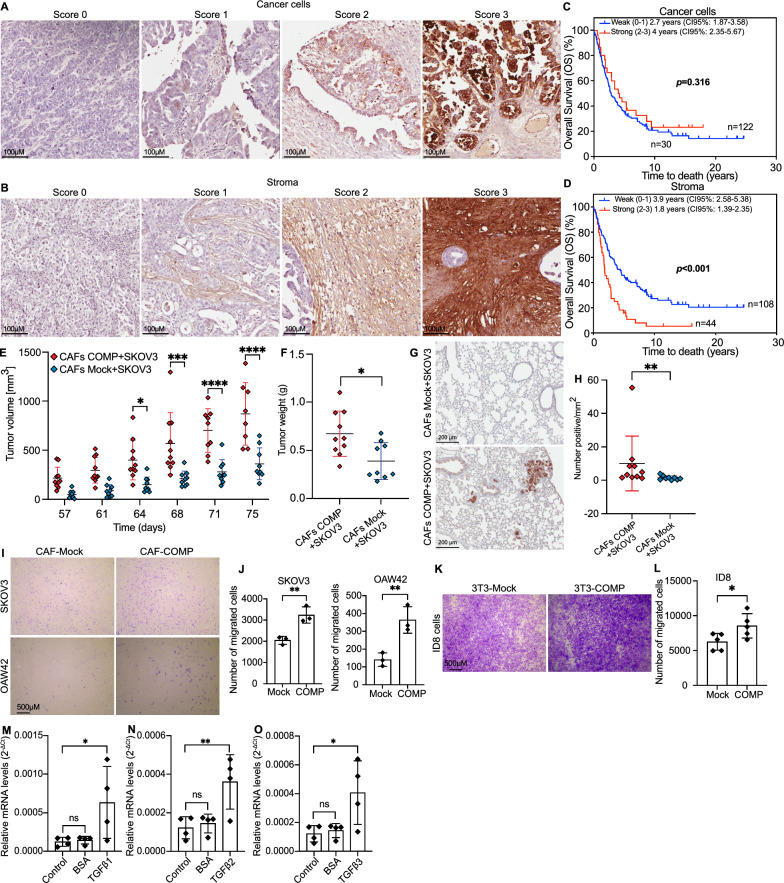
Elevated COMP expression in the stroma of ovarian cancer tumors drives survival and metastasis. Tissue microarrays of ovarian cancer patients were immunohistochemically stained to evaluate the expression of COMP **A** in tumor cells and **B** stroma. **C, D** For data analysis, COMP intensity was scored and samples were grouped as weak (scores 0 and 1) or strong (scores 2 and 3) COMP expression. Scale bar: 100 µm. Kaplan–Meier analysis was applied to examine overall survival according to high and low COMP expression in **C** tumor cells and **D** stroma. In vivo, NXG mice were co-injected by either COMP-expressing CAFs (COMP) or mock-CAFs (Mock) together with SKOV3 cells, respectively. **E** Tumor volumes (mm^3^) were evaluated weekly and statistical significance was calculated with two way-ANOVA followed by Sidak’s post-test. **F** Tumor weight was measured at the end of the experiment, and the *p*-value was calculated by the Mann–Whitney test. **G, H** Immunohistochemistry analysis of pan-cytokeratin expression in lungs derived from mice co-injected with COMP-expressing CAFs and SKOV3 cells versus Mock CAFs combined with SKOV3 cells. The *p*-value was calculated by the Mann–Whitney test. Scale bar: 200 µm. **I** The effect of secreted COMP by CAFs on the migration ability of ovarian cancer cell lines, SKOV3 and OAW42 **J**, was evaluated by transwell migration co-culture assays. **K, L** The same assay was performed using COMP-expressing 3T3 cells and ID8 cells. The *p*-value was calculated using an unpaired t-test. Scale bar: 500 µm. **M–O** CAF cells were treated with TGF-β isoforms (10 ng/ml) and *COMP* transcriptional expression was evaluated by RT-qPCR. PBS and BSA-treated cells were also included as a control. *HPRT1* was used as a reference gene, and the relative mRNA expression was calculated using the 2^−ΔCt^ method. The *p*-value was calculated by the One-way ANOVA followed by Dunnett’s post-test. *P* < 0.05 was considered statistically significant. (*, **, ***, and ns indicate *p* < 0.05, *p* < 0.01, *p* < 0.001, and non-significant, respectively.) Data are representative of at least three independent experiments and graphs depict the mean with the standard deviation

Immunohistochemical analyses showed varying degrees of COMP expression in ovarian cancer tissues (Fig. 1A, B), with high COMP expression in the stroma being strongly associated with decreased OS of ovarian cancer patients (*p* < 0.001) (Fig. 1D). Surprisingly, this association was not observed for COMP expressed by cancer cells (*p* = 0.316) (Fig. 1C). Notably, COMP expression by the cancer cells was correlated with the expression of COMP in stroma (n = 152, *p* < 0.001, Spearman's *ρ* = *0.503*). In addition, the overall expression of COMP in serous ovarian cancer, derived from the online Kaplan–Meier plotter database, indicated a correlation of high COMP expression with shorter OS and progression-free survival (Additional file 1: Fig S1 A, B) [30]. Moreover, as shown in Table 1, stromal COMP expression was significantly higher in the serous subtype (*p* = 0.005), in tumors of more advanced clinical FIGO (Federation Internationale de Gynecolgie et d'Obstetrique) stages (*p* = 0.005), and correlated significantly with high Ki67 expression (*p* = 0.019). On the contrary, there were no significant correlations of COMP expression in cancer cells with any clinicopathological parameters (Table 1). Multivariable Cox regression analysis (Table 2) showed that stronger expression of COMP in the stroma (*p* < 0.001, HR: 2.03, 95% CI 1.33–3.1) and more advanced clinical FIGO stages (*p* < 0.001, HR: 2.51, 95% CI 1.52–4.13) were independent predictors of shorter OS, whereas COMP expression in cancer cells was not prognostic. Furthermore, matched benign-appearing fallopian tube samples were also available for 37 patients in the cohort. The majority of these samples (86.5%) did not express COMP. Thus, when the paired samples (fallopian tubes / tumor samples) were analyzed with McNemar test, a gain of COMP expression was detected (*p* < 0.001) in the majority of tumor samples (Table 3). These results highlight the potential importance of COMP expression in the TME of epithelial ovarian cancer, likely secreted by CAFs, which are the primary cellular components of the stroma. Moreover, analysis of tumor tissues of high-grade serous tubo-ovarian cancer derived from the ScPanStroma database indicated that fibroblasts were the main stromal cell type expressing COMP (Additional file 1: Fig S1C) [31]. Furthermore, the expression of COMP in the stroma could serve as a promising prognostic indicator for ovarian cancer patients.

**Table 1:** Associations between COMP expression in stroma and cancer cells and clinicopathological characteristics

Factor	Patients N (%)	COMP intensity, stroma				*p-*value	COMP intensity, tumor				*p-*value
		0	1	2	3		0	1	2	3	
All	152 (100)	70 (45.5)	38 (24.7)	26 (16.9)	18 (11.7)		66 (43.4)	56 (36.8)	16 (10.5)	12 (7.9)	
Age	152 (100)										
< 55	28 (18.2)	13 (46.4)	6 (21.4)	7 (25.0)	2 (7.1)	0.211^a^	8 (28.6)	14 (50.0)	2 (7.1)	3 (10.7	0.110^a^
55–70	87 (56.5)	41 (47.1)	20 (23.0)	15 (17.2)	11 (12.6)		37 (42.5)	32 (36.8)	12 (13.8)	5 (5.7)	
> 70	37 (24.0)	16 (43.2)	12 (32.4)	4 (10.8)	5 (13.5)		21 (56.8)	10 (27.0)	2 (5.4)	4 (10.8)	
Subtype	152 (100)										
Serous	89 (58.6)	34 (37.8)	22 (24.4)	18 (20.0)	15 (16.7)	**0.005**^b^	33 (37.1)	37 (41.6)	12 (13.5)	6 (13.0)	0.108^b^
Non-Serous	63 (41.4)	36 (56.3)	16 (25.0)	8 (12.5)	3 (4.7)		33 (52.4)	19 (30.2)	4 (6.3)	6 (5.7)	
Grade	152 (100)										
1–2	106 (69.7)	47 (43,9)	25 (23.4)	21 (19.6)	13 (12.1)	0.360^b^	50 (47.2)	39 (36.8)	11 (10.4)	6 (5.7)	0.130^b^
3	46 (30.3)	23 (48.9)	13 (27.7)	5 (10.6)	5 (10.6)		16 (34.8)	17 (37.0)	5 (10.9)	6 (13.0)	
FIGO stage	138 (90.7)										
I	26 (17.1)	14 (53.8)	8 (30.8)	3 (11.5)	1 (3.8)	**0.005**^a^	14 (53.8)	4 (15.4)	2 (7.7)	6 (23.1)	0.522^a^
II	18 (11.8)	11 (61.1)	3 (16.7)	1 (5.6)	3 (16.7)		9 (50.0)	6 (33.3)	1 (5.6)	2 (11.1)	
III	73 (48.0)	30 (40.5)	21 (28.4)	15 (20.3)	7 (9.5)		32 (43.8)	31 (42.5)	7 (9.6)	2 (2.7)	
IV	21 (13.8)	6 (27.3)	4 (18.2)	4 (18.2)	7 (31.8)		6 (28.6)	10 (47.6)	3 (14.3)	2 (9.5)	
Ki67	150 (98.7)					**0.019**^a^					0.664^a^
0–10%	35 (23.0)	18 (51.4)	10 (28.6)	6 (17.1)	1 (2.9)		12 (34.3)	14 (40.0)	4 (11.4)	3 (8.6)	
11–25%	47 (30.9)	23 (48.9)	14 (29.8)	7 (14.9)	3 (6.4)		25 (53.2)	12 (25.5)	5 (10.6)	5 (10.6)	
> 25%	68 (44.7)	27 (39.7)	14 (20.6)	13 (19.1)	14 (20.6)		29 (42.6)	28 (41.2)	7 (10.3)	4 (5.9)	

Bold values indicate *p* < 0.05

COMP: cartilage oligomeric matrix protein, FIGO: Federation Internationale de Gynecolgie et d'Obstetrique. ^a^ Spearman correlation, two-tailed *p*-value. ^b^Mann–Whitney, two-tailed Exact *p*-value

**Table 2:** Cox multivariable-regression analyses of overall survival in relation to COMP expression in stroma and cancer cells

Overall Survival	Stroma			Cancer cells		
Variable	HR	95% CI	*p*-value	HR	95% CI	*p*-value
COMP (Weak vs strong)	2.03	1.33–3.10	**< 0.001**	1.03	0.63–1.70	0.906
Subtype (Non-Serous vs Serous)	1.39	0.90–2.15	0.140	1.47	0.95–2.28	0.086
Grade (1–2 vs 3)	0.63	0.39–1.01	0.053	0.66	0.41–1.05	0.082
FIGO stage (I-II vs III-IV)	2.51	1.52–4.13	** < 0.001**	2.63	1.58–4.37	**< 0.001**
Ki67 (0–10% vs 11–25% vs > 25%)	1.06	0.81–1.38	0.683	1.07	0.81–1.41	0.645

Bold values indicate *p*-values < 0.05

COMP: cartilage oligomeric matrix protein, FIGO: Federation Internationale de Gynecolgie et d'Obstetrique

**Table 3:** Alteration in COMP expression from benign-appearing fallopian tubes to the corresponding tumor

N = 37	Tumor samples				
	COMP negative		COMP positive		*p*-value
	N	%	N	%	
Fallopian tubes					**< 0.001**
COMP negative	12	32.4	20	54.1	
COMP positive	2	5.4	3	8.1	

Bold value indicate *p*-values < 0.05 calculated with McNemar analyses

COMP, cartilage oligomeric matrix protein

### COMP secretion by CAFs induces ovarian cancer cells tumorigenesis

To validate our hypothesis regarding the pivotal role of COMP in the TME, we first investigated the role of COMP expression by CAFs in vivo. In a xenograft mouse model, the co-injection of COMP-expressing CAFs at a 3 to 1 ratio with SKOV3 cells demonstrated a remarkable escalation in tumor volumes in comparison to the co-injection of mock-transfected CAFs with SKOV3 cells (Fig. 1E). By the end of the experiment, the tumor weight in mice co-injected with COMP-expressing CAFs was twice as high as that of tumors formed in mice co-injected with mock CAFs (Fig. 1F). We also observed pan-cytokeratin positive metastases in the lungs of both COMP and mock groups, with a higher prevalence in the COMP group compared to the mock group (Fig. 1G, H).

The migration ability of ovarian cancer cell lines, SKOV3 and OAW42, co-cultured with the COMP-expressing CAFs, was increased compared to the same ovarian cancer cell lines co-cultured with the mock CAFs (Fig. 1I, J). In this assay, SKOV3 or OAW42 cells were seeded in the upper layer of a cell culture insert with 8 µm pore size, while COMP-expressing CAFs or mock CAFs were cultured in the accompanied 24-well plate (Additional file 1: Fig. S2A). Consistently, COMP secreted by COMP-expressing 3T3 cells (mouse fibroblasts) significantly increased the migration ability of ID8 cells (mouse ovarian cancer cell line) compared with the control (Fig. 1K, L). This effect was observed without a direct interaction between the CAFs and ovarian cancer cells. Therefore, in alignment with the in vivo observations, these results emphasize the substantial paracrine influence of COMP-expressing CAFs on promoting ovarian cancer cells’ metastatic ability. Then, we hypothesized that the COMP expression by CAFs could be induced by the presence of the TGF-β in TME. In advanced tumor stages, TGF-βs exert major roles in TME, triggering tumor progression [32]. Moreover, it has been reported that there is a feedback loop between TGF-β and COMP in fibroblasts. Specifically, TGF-β treatment leads to COMP expression, which subsequently modulates TGF-β signaling [33]. In addition, a positive correlation between the expression of *COMP* and *TGFB* isoforms in ovarian serous cystadenocarcinoma was observed using the online cBioPortal and the TCGA database analyzed by the firehose legacy (Additional file 1: Fig S1D) [34, 35]. A similar correlation was also observed in ovarian tumors in the GEPIA2 database (Additional file 1: Fig S1E) [36]. Our RT-qPCR analysis also revealed a significant increase in *COMP* mRNA levels in CAFs treated with 10 ng/mL of TGF-β1, 2, & 3 for four days (Fig. 1M–O, Additional file 1: Fig. S2B). These results suggest that COMP secretion by CAFs, likely mediated by TGF-β, induces ovarian cancer cell tumorigenesis.

### COMP binds to the surface of the ovarian cancer cell lines and induces their migration and invasion

**Fig. 2:**
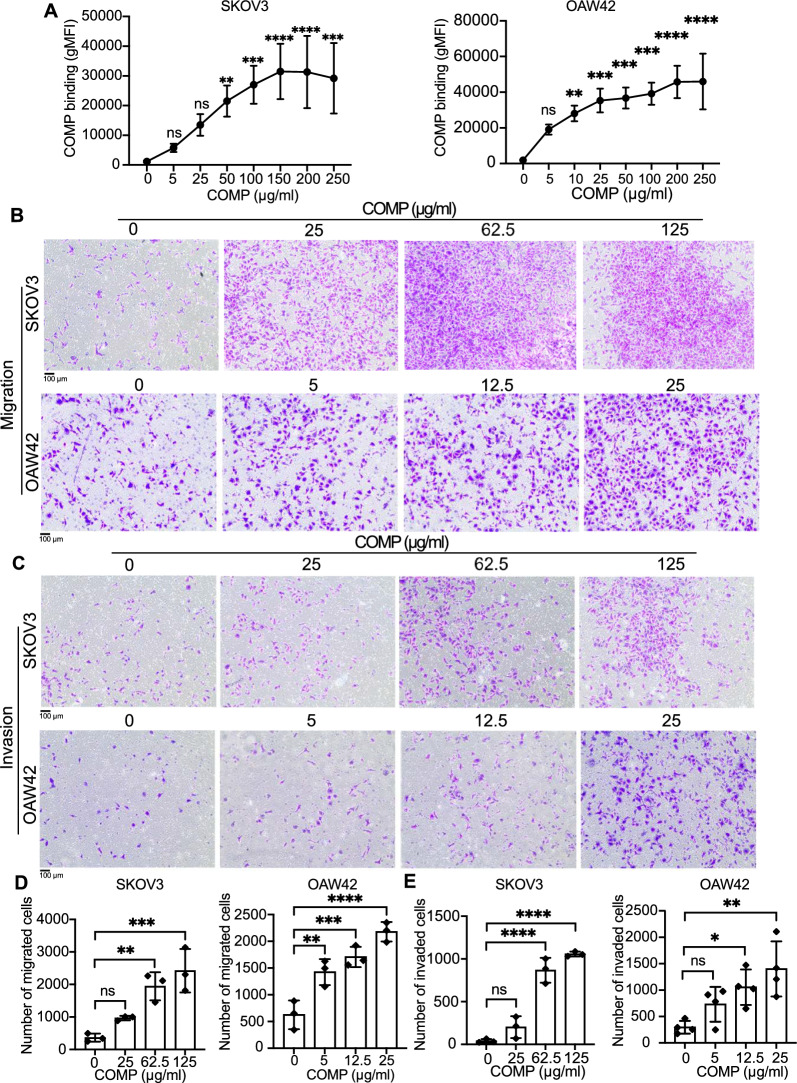
COMP binds to ovarian cancer cells and enhances their migration and invasion. **A** The binding ability of purified recombinant COMP to the surfaces of SKOV3 and OAW42 cells was evaluated by flow cytometry. Each concentration was compared with untreated cells as a control. Geometric mean fluorescence intensity: gMFI. The effect of increasing concentrations of COMP on SKOV3 and OAW42 on cell migration **B** and **D** and invasion **C** and **E** was assessed using transwell assays. The *p*-value was calculated by one-way ANOVA followed by Dunnett’s post-test. Data are representative of at least three independent experiments and graphs depict the mean with the standard deviation. Scale bar: 100 µm. *P* < 0.05 was considered statistically significant. (*, **, ***, ****, and ns indicate *p* < 0.05, *p* < 0.01, *p* < 0.001, *p* < 0.0001, and non-significant, respectively)

To further assess the paracrine effect of COMP on ovarian cancer cells, we first evaluated the binding capability of recombinantly expressed and purified COMP to ovarian cancer cell lines. A dose-dependent binding of recombinant COMP to the surfaces of SKOV3 and OAW42 cells was observed utilizing flow cytometry (Fig. 2A). Furthermore, the transwell migration and matrigel invasion assays showed a notable dose-dependent increase in the migration and invasion capabilities of SKOV3 and OAW42 cells when exposed to recombinant COMP, compared to the negative controls (Fig. 2B–E). In contrast, the cell proliferation assay revealed no significant difference in the proliferation of ovarian cancer cells in the presence of recombinant COMP (Additional file 1: Fig. S2C, D). Similarly, COMP secreted by CAFs did not affect the proliferation of CFSE-labeled SKOV3 cells, compared to mock control CAFs, in a direct coculture assay analyzed by flow cytometry (Additional file 1: Fig. S2E). These findings suggest that COMP binds to the surface of ovarian cancer cells, initiating migration and invasion—a characteristic of enhanced metastatic potential in a primary tumor.

### COMP induces ovarian cancer stem cells (CSCs) and tumor sphere formation

**Fig. 3:**
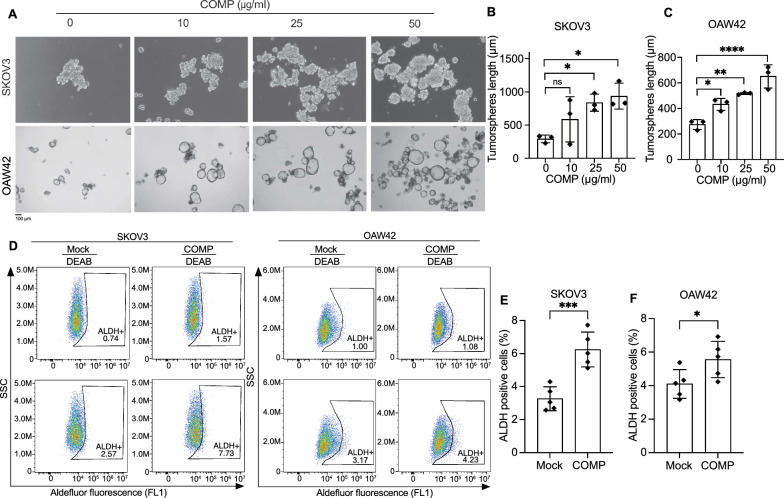
COMP induces ovarian cancer cells to form larger tumorspheres and a higher cancer stem cells population. Representative images **A** and length measurements **B, C** of tumorspheres formed by SKOV3 and OAW42 cells treated with increasing concentrations of COMP or PBS as a control. A minimum of ten spheroids per well was measured. The *p*-value was calculated by one-way ANOVA followed by Dunnett’s post-test. Scale bar: 100 µm. **D** The percentage of ALDH-expressing cells was evaluated in ovarian cancer cell lines **E** SKOV3 and **F** OAW42 utilizing the ALDEFLUOR assay, both in the presence or absence of COMP (20 μg/ml). ALDH inhibitor, DEAB, was used as a control for background fluorescence. The *p*-value was calculated by unpaired t-test. The data are representative of at least three independent experiments and graphs depict the mean with the standard deviation. *P* < 0.05 was considered statistically significant. (*, **, ***, ****, and ns indicate *p* < 0.05, *p* < 0.01, *p* < 0.001, *p* < 0.0001, and non-significant, respectively; ALDH: aldehyde dehydrogenase, DEAB: N, N-diethylaminobenzaldehyde)

Since no effect of COMP on the proliferation of ovarian cancer cells was observed, we examined the potential impact of COMP on CSC induction [13]. Evidence shows that tumor spheres can enrich CSCs and reflect their characteristics [37]. The effect of recombinant COMP on the CSCs population of ovarian cancer cell lines was assessed by the tumor sphere formation assay. Larger tumor spheres were formed by ovarian cancer cell lines, SKOV3 and OAW42, treated with recombinant COMP in a dose-dependent manner compared with control cells (Fig. 3A–C). To further confirm the effect of COMP on CSCs, the expression of aldehyde dehydrogenase (ALDH), a well-established ovarian CSCs marker [38], was assessed. SKOV3 and OAW42 cells were treated with recombinant COMP, and the expression of ALDH was evaluated by flow cytometry. A significant increase in ALDH-positive ovarian cancer cells was observed when cells were exposed to recombinant COMP for 48 h (Fig. 3D–F). These results demonstrate that COMP promotes CSCs in ovarian cancer cells.

### COMP increases the expression and activation of the Notch3 receptor by enhancing the Notch3 and Jagged1 interaction in ovarian cancer cell lines

**Fig. 4:**
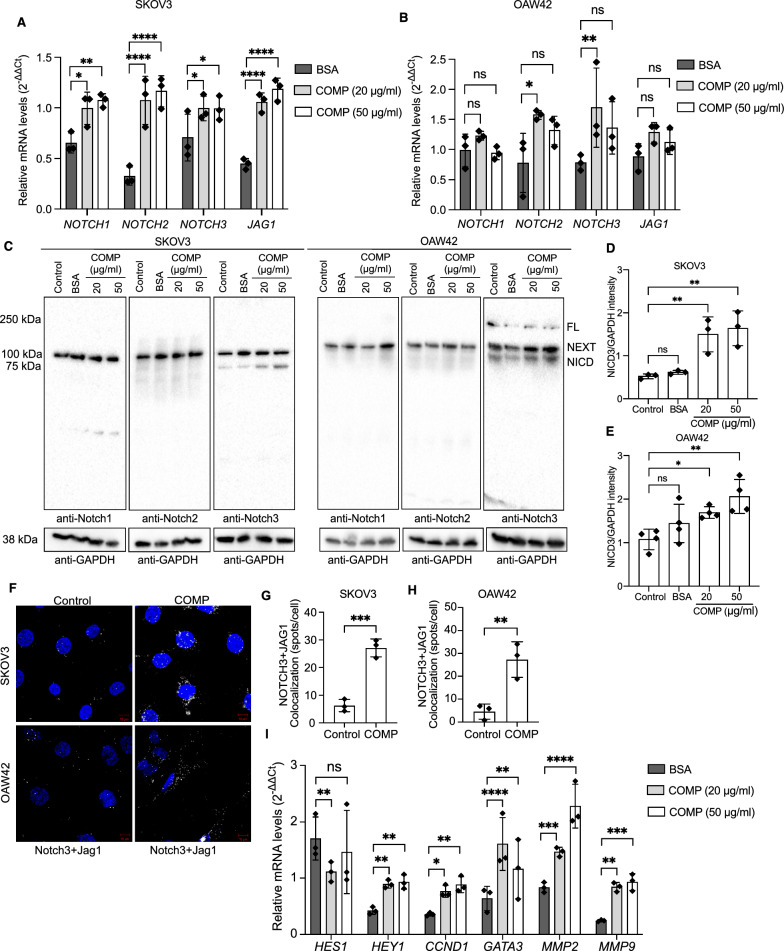
COMP activates Notch3 receptor and interacts with Jagged1 ligand. Analyses of the mRNA expression levels of *NOTCH1*, *NOTCH2*, *NOTCH3*, and *JAG1* in **A** SKOV3 and **B** OAW42 cells treated with different concentrations of COMP (20 and 50 μg/ml) or BSA (50 μg/ml) as a control by RT-qPCR. The *p*-value was calculated by two-way ANOVA followed by Tukey’s post-test. **C** The activation of Notch receptors in response to COMP (20 and 50 μg/ml) was assessed by western blot analyses in SKOV3 and OAW42 cells. PBS-treated and BSA (50 μg/ml)-treated cells were used as negative controls. **D, E** The protein expression levels of NICD3 were quantified by densitometry and normalized to GAPDH. One-way ANOVA followed by Dunnett’s post-test was used for statistical analyses. **F** The colocalization of Notch3 receptor and its ligand Jagged1 was evaluated in response to COMP (0.25 mg/ml) by PLA assay in **G** SKOV3 and **H** OAW42 cells (Blue areas: DAPI-stained nuclei, white dots: Notch3-Jagged1 co-expression). The *p*-value was calculated by unpaired t-test. **I** The mRNA expression levels of Notch target genes were evaluated in SKOV3 cells treated with different concentrations of COMP (20 and 50 μg/ml) or BSA (50 μg/ml) as a control by RT-qPCR. The *p*-value was calculated by two-way ANOVA followed by Dunnett’s post-test. *ACTB* was used as a reference gene, and the relative mRNA expression was calculated using the 2^−ΔΔ^^Ct^ method. The data are representative of at least three independent experiments and graphs depict the mean with the standard deviation. *P* < 0.05 was considered statistically significant. (*, **, ***, ****, and ns indicate *p* < 0.05, *p* < 0.01, *p* < 0.001, *p* < 0.0001, and non-significant, respectively; NICD: Notch intracellular domain, NEXT: Notch extracellular truncated domain, FL: Full length)

RT-qPCR analyses revealed a statistically significant upregulation of Notch receptors following treatment with recombinant COMP. *NOTCH1*, *NOTCH2*, *NOTCH3*, and *JAG1* were upregulated upon stimulation with COMP. Particularly, a significant upregulation of all tested genes was observed for both concentrations of COMP (20 and 50 μg/ml) in SKOV3 cells (Fig. 4A), while significant upregulation was only observed for *NOTCH2* and *NOTCH3* expression in OAW42 cells treated with 20 μg/ml of COMP (Fig. 4B). Thereafter, Notch activation in response to recombinant COMP was evaluated. Upon binding of Notch receptors to their specific ligands on the surface of adjacent cells, the intracellular domain of Notch receptors undergoes proteolytic cleavage by α- and γ-secretases, followed by translocation into the nucleus of the signal-receiving cell [39]. This domain can be detected as a distinct band in Western blot analysis. The western blotting results indicated that only Notch3 was activated in SKOV3 and OAW42 cells treated with 20 μg/ml and 50 μg/ml of recombinant COMP (Fig. 4C). Although the mRNA levels of *NOTCH1* and *NOTCH2* were increased in COMP-treated cells, the western blot results showed that Notch1 and Notch2 remained inactive in the presence of COMP. Furthermore, the intensity analyses of Notch3 intracellular domain (NICD3) normalized to GAPDH showed a significant increase in NICD3 in both ovarian cancer cell lines treated with recombinant COMP, compared with untreated cells as a control, indicating the specific activation of the Notch3 receptor upon COMP treatment (Fig. 4D, E).

To elucidate the molecular mechanism of Notch3 activation by COMP, we examined the effect of recombinant COMP on the interaction between Notch3 and its ligand Jagged1 using a proximity ligation assay (PLA). The results revealed a higher number of spots per cell in COMP-treated ovarian cancer cells compared to the control group, indicating an increased interaction of Notch3 and Jagged1 in the presence of COMP (Fig. 4F–H). Consistently, we observed an increased expression of Notch3 downstream target genes, such as *HEY1* (hes related family bHLH transcription factor with YRPW motif 1), *CCND1* (cyclin D1), *GATA3* (GATA binding protein 3), *MMP2* (matrix metallopeptidase 2), and *MMP9* (matrix metallopeptidase 9) in COMP-treated SKOV3 cells at mRNA level (Fig. 4I). These compelling observations show that COMP selectively activates the Notch3 signaling pathway in ovarian cancer cells.

### COMP-induced migration and tumor sphere formation are Notch-dependent

**Fig. 5:**
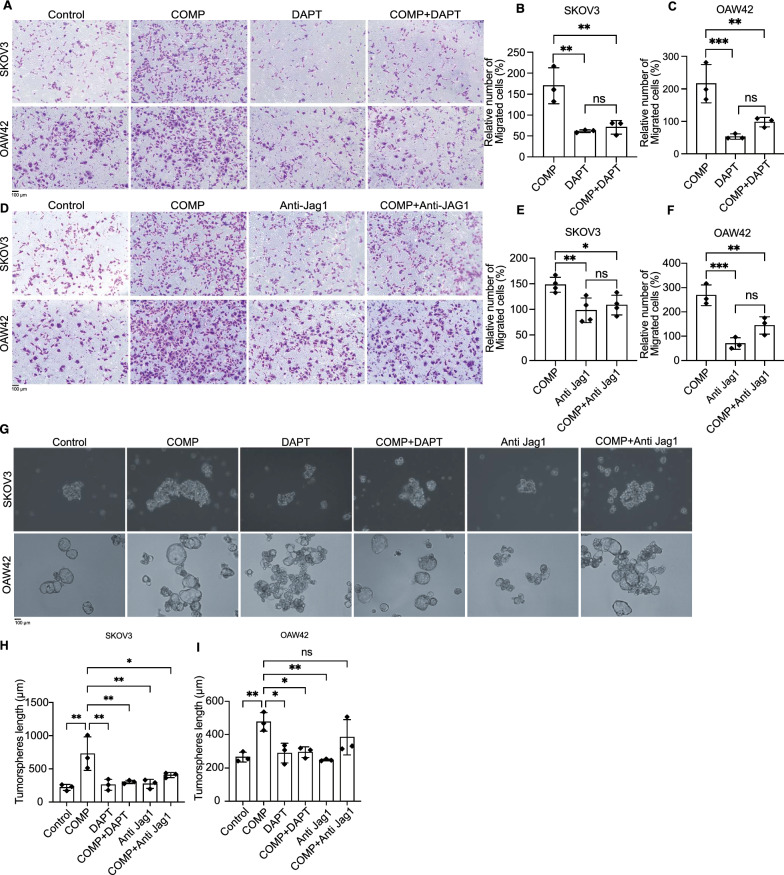
Notch inhibitors diminish the COMP-induced migration and tumorspheres size of ovarian cancer cells. Transwell migration assays for SKOV3 and OAW42 cells treated with COMP (COMP), DAPT (10 μM), and COMP in combination with DAPT (COMP + DAPT). **A** Representative images and **B, C** the number of migrated cells in the presence of DAPT. The number of migrated cells was normalized to the number of control cells (PBS-treated). **D–F** The same experiments were performed using an anti-Jagged1 antibody (5 μg/ml) in the presence or absence of COMP. In both experiments, 62.5 μg/ml and 12.5 μg/ml of COMP were used for SKOV3 and OAW42 cells treatment, respectively. PBS-treated cells were also used as a control. **G–I** Tumorsphere formation assays were performed for SKOV3 and OAW42 cells treated with COMP (20 μg/ml), DAPT (1 μM), and anti-Jagged1 antibody (2 μg/ml). PBS-treated cells were used as a control. A minimum of 10 spheroids per well were measured. Scale bar: 100 µm. The *p*-value was calculated by one-way ANOVA followed by Tukey’s post-test. The data are representative of at least three independent experiments, and graphs depict the mean with the standard deviation. *P* < 0.05 was considered statistically significant. (*, **, ***, ****, and ns indicate *p* < 0.05, *p* < 0.01, *p* < 0.001, *p* < 0.0001, and non-significant, respectively)

To confirm the functional association of COMP with the Notch signaling pathway, we assessed the effect of Notch inhibitors, DAPT (γ-secretase inhibitor), and an anti-Jagged1 antibody on the COMP-induced migration and tumorsphere formation. Transwell migration assays indicated that COMP-induced migration was significantly reduced by DAPT (10 μM; Fig. 5A–C) and the anti-Jagged1 antibody (5 μg/ml) in both ovarian cancer cell lines (Fig. 5D–F). The tumor sphere formation assays also revealed a reduction in spheroid size upon treatment with DAPT (1 μM) or anti-Jagged1 antibody (2 μg/ml) in combination with COMP (20 μg/ml), compared to COMP-treated cells, for SKOV3 (COMP Vs. COMP + DAPT *p* < 0.01, COMP Vs. COMP + Anti Jagged1 *p* < 0.05) and OAW42 cells (COMP Vs. COMP + DAPT *p* < 0.05) (Fig. 5G-I). These results suggest that the effect of COMP on migration and tumor sphere formation of ovarian cancer cells is Notch dependent.

### COMP induces the EMT in ovarian cancer cells

**Fig. 6:**
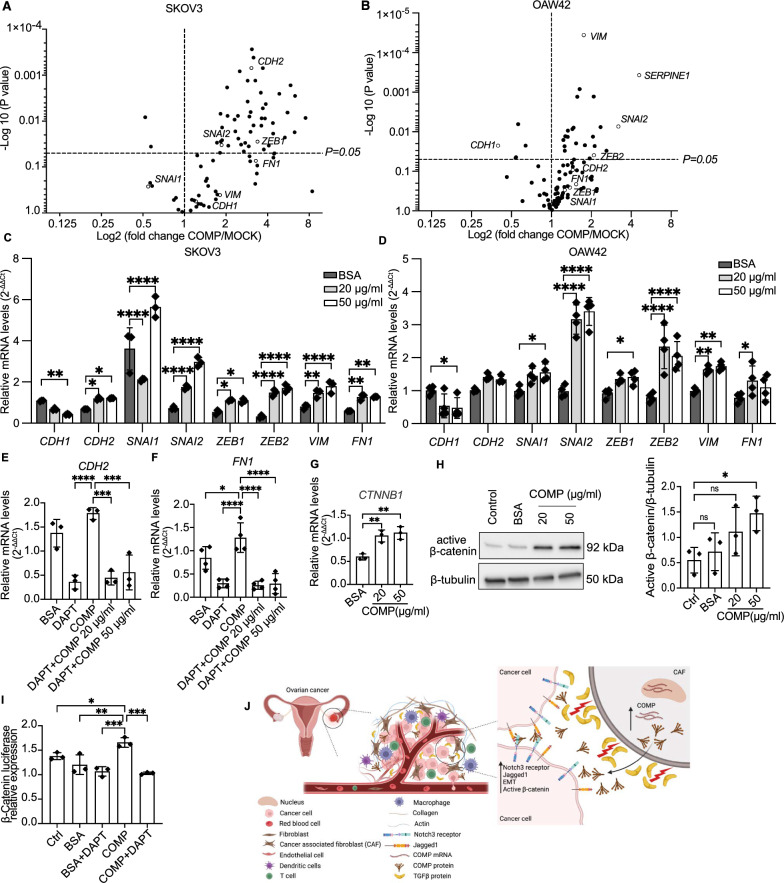
COMP induces EMT and cross-talks with β-catenin signaling pathway. The mRNA expression levels of EMT-specific genes in **A** SKOV3 and **B** OAW42 cells treated with COMP (20 μg/ml) or BSA (20 μg/ml) were analyzed using a PrimePCR Custom Plate. Volcano plots with a Log2 scale on the X-axis and a Log10 scale on the Y-axis were used to represent the data. *GAPDH* was used as a reference gene, and the relative mRNA expression was calculated using the 2^−ΔΔCt^ method. Multiple unpaired t-tests were used for statistical analyses. **C, D** RT-qPCR analysis of EMT marker genes, in SKOV3 and OAW42 cells treated with COMP (20 and 50 μg/ml) or BSA (50 μg/ml) as a control. *GAPDH* was used as a reference gene, and the relative mRNA expression was calculated using the 2^−ΔΔCt^ method. The *p*-value was calculated by two-way ANOVA followed by Tukey’s post-test. **E, F** RT-qPCR analysis of *CDH2* and *FN1* expression in SKOV3 cells treated with DAPT (1 μM), COMP (50 μg/ml), and DAPT + COMP 20 μg/ml and 50 μg/ml. *GAPDH* was used as a reference gene and the relative mRNA expression was calculated using the 2^−ΔΔCt^ method. The *p*-values were calculated by one-way ANOVA followed by Dunnett’s post-test. **G** RT-qPCR analysis of *CTTNB1* (β-catenin) in SKOV3 cells in the presence of COMP (20 and 50 μg/ml) or BSA (50 μg/ml) as a control. *GAPDH* was used as a reference gene, and the relative mRNA expression was calculated using the 2^−ΔΔCt^ method. The *p*-value was calculated by one-way ANOVA followed by Tukey’s post-test. **H** Analysis of protein expression levels of active (non-phosphorylated) β-catenin in SKOV3 cells treated with COMP (20 and 50 μg/ml) or BSA (50 μg/ml) as a control by western blotting. The *p*-value was calculated by one-way ANOVA followed by Dunnett’s post-test. **I** Dual-Luciferase reporter assay measuring the activity of β-catenin in SKOV3 cells treated with BSA (50 μg/ml), BSA + DAPT (1 μM), COMP (50 μg/ml), and COMP + DAPT (1 μM). Untreated cells were used as a control. The *p*-value was calculated by one-way ANOVA followed by Dunnett’s post-test. **J** Graphical representation of the proposed molecular mechanism of COMP paracrine action. Stromal expression of COMP likely by CAFs in ovarian TME bridges Notch3-jagged1 interaction, leading to EMT induction and β-catenin activation. The data are representative of at least three independent experiments and graphs depict the mean with the standard deviation. The significance threshold was set at *p* < 0.05. (*, **, ***, ****, and ns indicate *p* < 0.05, *p* < 0.01, *p* < 0.001, *p* < 0.0001, and non-significant, respectively)

Growing evidence indicates that the activation of EMT by Notch signaling pathway is associated with cancer aggressiveness [40]. RT-qPCR array for EMT-specific genes showed that the expression of seventeen genes was significantly changed in both ovarian cancer cell lines upon stimulation with COMP (Fig. 6A, B, Additional file 1: Table S2 and S3). Furthermore, RT-qPCR analysis confirmed that the mRNA level of the epithelial marker *CDH1* (E-cadherin), was significantly downregulated in response to 50 μg/ml COMP for both cell lines, whereas mesenchymal markers, including *CDH2* (N-cadherin), *VIM* (vimentin), and *FN1* (fibronectin 1), as well as EMT transcription factors including *SNAI1* (snail family transcriptional repressor 1), *SNAI2* (snail family transcriptional repressor 2), *ZEB1* (zinc finger E-box binding homeobox 1), and *ZEB2* (zinc finger E-box binding homeobox 2), were upregulated (Fig. 6C, D). However, the upregulation of *CDH2* was not statistically significant in OAW42 cells (Fig. 6D). Moreover, the expression of *CDH2* and *FN1* were decreased by DAPT (1 μM), signifying the NOTCH dependency of COMP effect on EMT (Fig. 6E, F). These data strongly indicate that stimulation with COMP induces EMT in ovarian cancer cell lines.

### Extracellular COMP activates the β-catenin signaling pathway

A noteworthy aspect of the Notch signaling pathway is its ability to cross-talk with other signaling pathways such as TGF-β, VEGF, Ras, PI3K/AKT, mTOR, and β-Catenin, resulting in the involvement of Notch in diverse physiological and pathological conditions [41]. Hence, we sought to investigate whether COMP affects the activity of the β-Catenin pathway, which is of great importance in oncogenesis. RT-qPCR analysis showed that β-Catenin mRNA levels were increased in COMP-treated SKOV3 cells compared with the control (Fig. 6G). Furthermore, at the protein level, non-phosphorylated active β-catenin was significantly overexpressed in the presence of 50 μg/ml COMP compared with the control in SKOV3 cells (Fig. 6H). However, there were no changes in the expression of total β-catenin and p-GSK3β (glycogen synthase kinase 3β) after COMP treatment (Additional file 1: Fig S2G). Moreover, using the dual-luciferase reporter assay indicated that COMP (50 μg/ml)-induced activation of β-catenin was inhibited in the presence of DAPT (1 μM), suggesting the Notch dependency of COMP function in activating β-catenin (Fig. 6I). Collectively, these results suggest that COMP can activate the β-catenin signaling pathway.

### Extracellular COMP does not protect ovarian cancer cells from apoptosis

It has been shown that the intracellular expression of COMP protects cells against apoptosis via disruption of calcium release from the endoplasmic reticulum [9], inhibition of active caspase-3 and overexpression of IAP family proteins [42]. To evaluate the potential protective role of recombinant COMP against apoptosis, through binding to the cell surface, in ovarian cancer cells, we conducted an Annexin V-Zombie aqua dye apoptosis assay, with cisplatin serving as the apoptosis inducer (Additional file 1: Fig. S2F). The results demonstrated that exposing the cisplatin-treated cells to recombinant COMP did not alter the number of apoptotic cells, suggesting that cell membrane-bound COMP does not confer protection against apoptosis in ovarian cancer cells.

## Discussion

In this study, we identified a significant correlation between increased COMP expression in tumor stroma and shorter OS in ovarian cancer patients. Consistently, mice co-transplanted with COMP-expressing CAFs and ovarian cancer cells exhibited considerably larger tumors and a higher frequency of lung metastases compared to the control group co-transplanted with mock CAFs and ovarian cancer cells. Mechanistically, the membrane-bound COMP induced EMT and cancer stemness via the activation of the Notch3 axis.

The TME consists of cellular components such as fibroblasts and immune cells, as well as non-cellular components like extracellular matrix proteins, growth factors, and chemokines. TME components such as COMP play major roles in cancer progression and aggressiveness, making them an attractive target for cancer therapeutics [43]. Growing evidence has unveiled associations of COMP expression with a worse outcome in several solid cancer types, including breast, prostate, colorectal, bladder, and hepatocellular carcinoma [9, 11, 12, 15, 44]. For example, COMP levels in the serum of metastatic breast cancer patients were correlated with metastases to the liver, brain, and lung, as well as decreased OS, rendering it an independent prognostic biomarker [16]. Further, COMP expression in cancer cells of breast cancer and intestinal-type periampullary adenocarcinoma was shown to be associated with a shorter overall and recurrence free survival of the patients. This association was also observed for COMP expression in the tumor stroma in intestinal-type periampullary adenocarcinoma, but not in breast cancer patients [11, 24]. In ovarian cancer, a high proportion of stromal cells, defined by the expression of *COL1A1*, was correlated with shorter survival rate of patients [45].

Interestingly, COMP has been shown to be the most overexpressed protein in the stroma of omental metastases of high-grade serous ovarian cancer patients [17], and higher in the stroma of omental biopsies than in tumor cells [46]. In our study, increased COMP expression in the ovarian tumor stroma correlated with shorter OS, serous histological subtype and more advanced clinical FIGO stages. These observations are consistent with the higher number of metastases observed in the animal model in this study. The discovery of a link between stromal COMP and adverse clinical outcomes motivated us to investigate how stromal COMP in the TME influences ovarian cancer cells, which might provide crucial insights into the mechanisms underlying ovarian cancer progression and metastasis.

In the stromal context, CAFs play critical roles as a tumor-promoting agent by secretion of signaling molecules such as growth factors and interleukins into the TME, direct physical interaction with cancer cells, and secretion of extracellular vesicles [47]. In vivo, co-injection of COMP-expressing CAFs and SKOV3 cells resulted in larger tumors than mock CAF cells combined with SKOV3 cells. Further, more lung metastases were observed in the COMP group compared to the mock group, confirming the metastasis-promoting effect of COMP secreted by CAFs in the ovarian TME. Consistent with previous studies, we found that TGF-β stimulates COMP expression in CAFs [46, 48]. TGF-β is one of the major growth factors in TME with recognized roles in tumor progression and can potentiate cell migration, tumor growth, and metastases [47]. In fact, the relation between COMP and TGF-β family members is reciprocal. For instance, it has been shown that the TGFB1-mediated signaling pathway is required for COMP expression by bone marrow-derived stem cells [49]. On the other hand, the binding of TGF-β1 dimer to the C-terminal domain of the COMP molecule forms a complex with TGF-β receptors, resulting in a higher engagement of TGF-β receptors in the presence of pentameric COMP molecules, which in turn enhances the cellular response to TGF-β1 [50].

To study COMP’s mechanism of action, we used purified recombinant COMP and observed a dose-dependent binding of COMP to ovarian cancer cells, which enhanced their migration and invasion capabilities. Similarly, recombinant COMP binding to prostate cancer cells was previously shown to enhance their migration and invasion, which was abolished by an integrin inhibitor, Cilengitide [9]. Evidence indicates that COMP may interact with fibronectin [51], CD47, and αVβ3 integrin on primary chondrocyte surfaces [52]. A similar characteristic has been assigned to other thrombospondin family members. For instance, recombinant TSP1 increases the wound healing, migration, and invasion of osteosarcoma cells through the FAK signaling pathway [53]. COMP did not significantly affect ovarian cancer cell proliferation in vitro, as also observed with breast and prostate cancer cells [9, 11], consistent with no observed correlation between COMP in cancer cells and Ki67 by immunochemistry analysis. In contrast, COMP promoted the proliferation of colon cancer and hepatocellular carcinoma cells [10, 15], highlighting the diverse mechanisms by which COMP acts in different cancer types. Moreover, in the present study, we found that the extracellular COMP did not protect ovarian cancer cells from apoptosis. A similar result was also observed in prostate cancer cells exposed to staurosporine and treated with recombinant COMP. In contrast, the intracellular expression of COMP protected cells against several apoptotic inducer agents, which was attributed to the impaired Ca^2+^ release from the endoplasmic reticulum in COMP-expressing cells [9]. These results suggest that the anti-apoptotic effect of COMP depends on its intracellular expression, a phenomenon that may implicate a novel role of COMP in cancer chemoresistance and would be of interest to investigate in future studies.

Since COMP did not affect the proliferation and apoptosis of ovarian cancer cells, we hypothesized that the larger primary tumors in mice co-injected with COMP-expressing CAFs observed in vivo might be due to the COMP effect on CSCs expansion. CSCs, a subtype of cancer cells, are able to differentiate into other cell types, self-renew, initiate new tumors, and potentiate cancer recurrence, drug resistance, and metastasis [54]. Previously, we showed that COMP secreted by breast cancer cells promotes the CSC population by activating the Notch3 signaling pathway [13]. Herein, a similar observation was made for ovarian cancer cells treated with recombinant COMP using tumorsphere formation assay. Hence, COMP in the TME may trigger ovarian CSCs and contribute to disease progression. Notch receptors physiologically regulate stemness and cellular fate, and cross-talk with other signaling pathways. Therefore, their aberrant expression is considered a pathological condition. Growing evidence suggests that the Notch signaling pathway displays both pro-inhibitory and pro-tumorigenic functions, greatly depending on the cancer type and the Notch receptor [55]. Previously, we showed that COMP associates with β-catenin and AKT pathways by activating the Notch3 receptor, whereby it bridges the Notch3-jagged1 interaction [13]. Herein, we further confirmed the role of COMP in mediating the interaction between Notch3 and Jagged1 using PLA. The proposed mechanism suggests that polymeric COMP functions as a bridge connecting the extracellular domain of Notch3 with Jagged-1 on the cell surface. The activation of Notch3 is not limited to COMP but can also be achieved by other TSP family members, such as TSP2 function in lung and ovarian cancer cells [56]. Notch triggers tumorigenesis, including tumor initiation, spheroid formation, proliferation, EMT, metastasis, and drug resistance [55, 57]. Consistently, in the present study, Notch dependency of the migration and tumorspheres formation induced by COMP in ovarian cancer cells was observed.

The transition from epithelial to mesenchymal status is another consequence of Notch activation involved in extracellular remodeling and increases the likelihood of cancer cell dissemination to adjacent or remote tissues. Therefore, we hypothesized that the paracrine effect of COMP on increasing the metastatic potential of ovarian cancer cells, observed in vivo, is mediated by the induction of EMT in ovarian cancer cells. Indeed, given the Notch3 activation by COMP, we subsequently observed EMT induction in ovarian cancer cells following COMP treatment. Mechanistically, upon Notch activation, its intracellular domain affects the EMT transcription factors, including ZEB1 and ZEB2, SNAI1 and 2, and TWIST1 and 2 in the nucleus, followed by upregulation of mesenchymal genes such as N-cadherin, vimentin, and fibronectin [58]. In support of these findings, COMP co-expression with mesenchymal markers was reported through bioinformatic analysis [12, 59]. In hepatocellular carcinoma, MEK and PI3K inhibitors abrogated the COMP-induced EMT, which was related to COMP interaction with CD36 receptors [15]. β-catenin is another signaling pathway affected by Notch intracellular domain, which in turn leads to EMT [58]. We previously observed that β-catenin expression was increased in COMP-expressing breast cancer cells, further enhanced by Notch inhibitors [13]. Herein, recombinant COMP treatment resulted in the overexpression of active non-phosphorylated β-catenin in SKOV3 cells, and Notch inhibition by DAPT abolished the expression of active β-catenin.

## Conclusions

Taken together, we found that high stromal COMP expression in ovarian tumors was associated with unfavorable clinicopathological characteristics and shorter patient survival. In vivo and in vitro experiments confirmed the paracrine role of COMP secreted by CAFs, potentially upregulated by TGF-βs, in driving the ovarian cancer cells’ aggressiveness. Mechanistically, membrane-bound COMP enhanced cell migration and ovarian CSC expansion in a Notch-dependent manner. Furthermore, COMP activated the Notch3 signaling pathway and upregulated active β-catenin and EMT markers in a Notch-dependent manner. These findings illuminate the pivotal role of COMP in ovarian cancer progression and offer valuable insights into its main source within the TME and the underlying mechanisms of action.

### Supplementary Information

**Additional file 1: Figure S1. A** and **B**Kaplan-Meier plots, obtained from the online KM plotter, representing the correlations between the higher expression of COMP with a shorter OS and progression-free survival of serous ovarian cancer patients. **C** Levels of *COMP* expression by stromal cells in high-grade serous tubo-ovarian cancer, extracted from ScPanStroma database. **D** The positive correlation between *COMP* and different isoforms of *TGFB* (*B1*, *B2*, and *B3*) in Ovarian Serous Cystadenocarcinoma, extracted from cBioPortal database. **E** The positive correlation between *COMP* and different isoforms of *TGFB* (*B1*, *B2*, and *B3*) in ovarian tumor dataset, extracted from GEPIA2 database using spearman correlation coefficient. OS: Overall survival, PFS: Progression-free survival. **Figure S2.** Graphical schemes representing the co-culture assay of COMP-expressing CAFs with ovarian cancer cell lines **A** and CAFs treatment with TGF-β isoforms **B**. **C, D** Cell proliferation assay for SKOV3 and OAW42 cells treated with increasing concentrations of COMP using CyQUANT cell proliferation assay kit. Data were normalized to PBS-treated cells. The *p*-value was calculated by one-way ANOVA followed by Dunnett’s post-test (n=3, mean±SD). **E.** Cell proliferation assay for CFSE-labeled SKOV3 cells cocultured with COMP-expressing CAFs or the mock control CAFs using flowcytometry. The *p*-value was calculated by two-way ANOVA followed by Sidak’s post-test (n=3, mean±SD). **F** Apoptosis assay using Annexin V-APC and Zombie aqua dye in four samples, including BSA (20 μg/ml)-treated, COMP (20 μg/ml)-treated, cisplatin-treated, and COMP in combination with cisplatin-treated (COMP+cisplatin) of SKOV3 and OAW42 cells. SKOV3 and OAW42 cells were treated with 10 μM and 20 μM of cisplatin, respectively. The *p*-value was calculated by one-way ANOVA followed by Sidak’s post-test (n=3, mean±SD). **G** Evaluation of total β-catenin and pGSK3β protein expression levels in SKOV3 cells treated with COMP (20 and 50 μg/ml) or BSA (50 μg/ml) as a control by western blotting. PBS-treated cells were also used as a control. β-tubulin served as a loading control. The *p*-value was calculated by one-way ANOVA followed by Dunnett’s post-test. The data are representative of three independent experiments and graphs depict mean±SD. (*, **, and ns indicate *p*<0.05, *p*<0.01, and non-significant, respectively). **Table S1.** List of the used antibodies. **Table S2.** Significant deregulated EMT-specific genes in SKOV3 cell line treated with recombinant COMP (20 μg/ml) or BSA (20 μg/ml) obtained by RT-qPCR array. **Table S3**: Significant deregulated EMT-specific genes in OAW42 cell line treated with recombinant COMP (20 μg/ml) or BSA (20 μg/ml) obtained by RT-qPCR array.

## Data Availability

The datasets generated during and/or analysed during the current study are available from the corresponding author on reasonable request.

## Abbreviations

COMP: Cartilage oligomeric matrix protein
OS: Overall survival
CAF: Carcinoma-associated fibroblast
TME: Tumor microenvironment
EMT: Epithelial-to-mesenchymal transition
CSC: Cancer stem cell
ALDH: Aldehyde dehydrogenase
