# Supplementary material for: Stromal cartilage oligomeric matrix protein as a tumorigenic driver in ovarian cancer via Notch3 signaling and epithelial-to-mesenchymal transition
Source: J Transl Med. 2024 Apr 13;22:351. doi: 10.1186/s12967-024-05083-0 (PMC11016227; doi:10.1186/s12967-024-05083-0)
Supplement: Supplementary file 1 — Additional file 1: Figure S1. A and BKaplan-Meier plots, obtained from the online KM plotter, representing the correlations between the higher expression of COMP with a shorter OS and progression-free survival of serous ovarian cancer patients. C Levels of COMP expression by stromal cells in high-grade serous tubo-ovarian cancer, extracted from ScPanStroma database. D The positive correlation between COMP and different isoforms of TGFB (B1, B2, and B3) in Ovarian Serous Cystadenocarcinoma, extracted from cBioPortal database. E The positive correlation between COMP and different isoforms of TGFB (B1, B2, and B3) in ovarian tumor dataset, extracted from GEPIA2 database using spearman correlation coefficient. OS: Overall survival, PFS: Progression-free survival. Figure S2. Graphical schemes representing the co-culture assay of COMP-expressing CAFs with ovarian cancer cell lines A and CAFs treatment with TGF-β isoforms B. C, D Cell proliferation assay for SKOV3 and OAW42 cells treated with increasing concentrations of COMP using CyQUANT cell proliferation assay kit. Data were normalized to PBS-treated cells. The p-value was calculated by one-way ANOVA followed by Dunnett’s post-test (n=3, mean±SD). E. Cell proliferation assay for CFSE-labeled SKOV3 cells cocultured with COMP-expressing CAFs or the mock control CAFs using flowcytometry. The p-value was calculated by two-way ANOVA followed by Sidak’s post-test (n=3, mean±SD). F Apoptosis assay using Annexin V-APC and Zombie aqua dye in four samples, including BSA (20 μg/ml)-treated, COMP (20 μg/ml)-treated, cisplatin-treated, and COMP in combination with cisplatin-treated (COMP+cisplatin) of SKOV3 and OAW42 cells. SKOV3 and OAW42 cells were treated with 10 μM and 20 μM of cisplatin, respectively. The p-value was calculated by one-way ANOVA followed by Sidak’s post-test (n=3, mean±SD). G Evaluation of total β-catenin and pGSK3β protein expression levels in SKOV3 cells treated with COMP (20 and 50 μg/ml) or BSA (50 μg/ml) a [file 12967_2024_5083_MOESM1_ESM.docx]

**
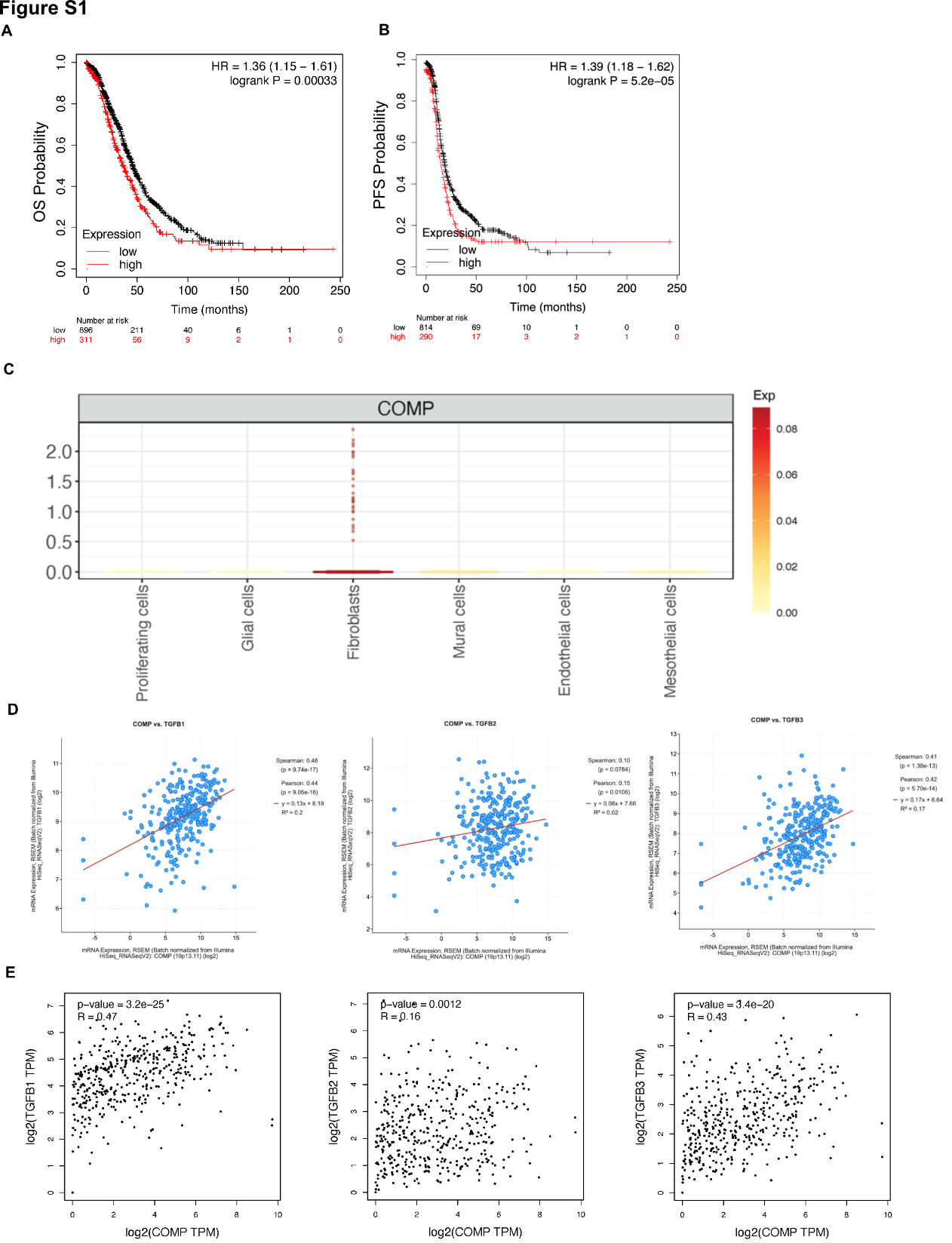
**

**Figure S1. A&B.** Kaplan-Meier plots, obtained from the online KM plotter, representing the correlations between the higher expression of COMP with a shorter OS and progression-free survival of serous ovarian cancer patients. **C.** Levels of *COMP* expression by stromal cells in high-grade serous tubo-ovarian cancer, extracted from ScPanStroma database. **D.** The positive correlation between *COMP* and different isoforms of *TGFB* (*B1*, *B2*, and *B3*) in Ovarian Serous Cystadenocarcinoma, extracted from cBioPortal database. **E.** The positive correlation between *COMP* and different isoforms of *TGFB* (*B1*, *B2*, and *B3*) in ovarian tumor dataset, extracted from GEPIA2 database using spearman correlation coefficient. OS: Overall survival, PFS: Progression-free survival.

**
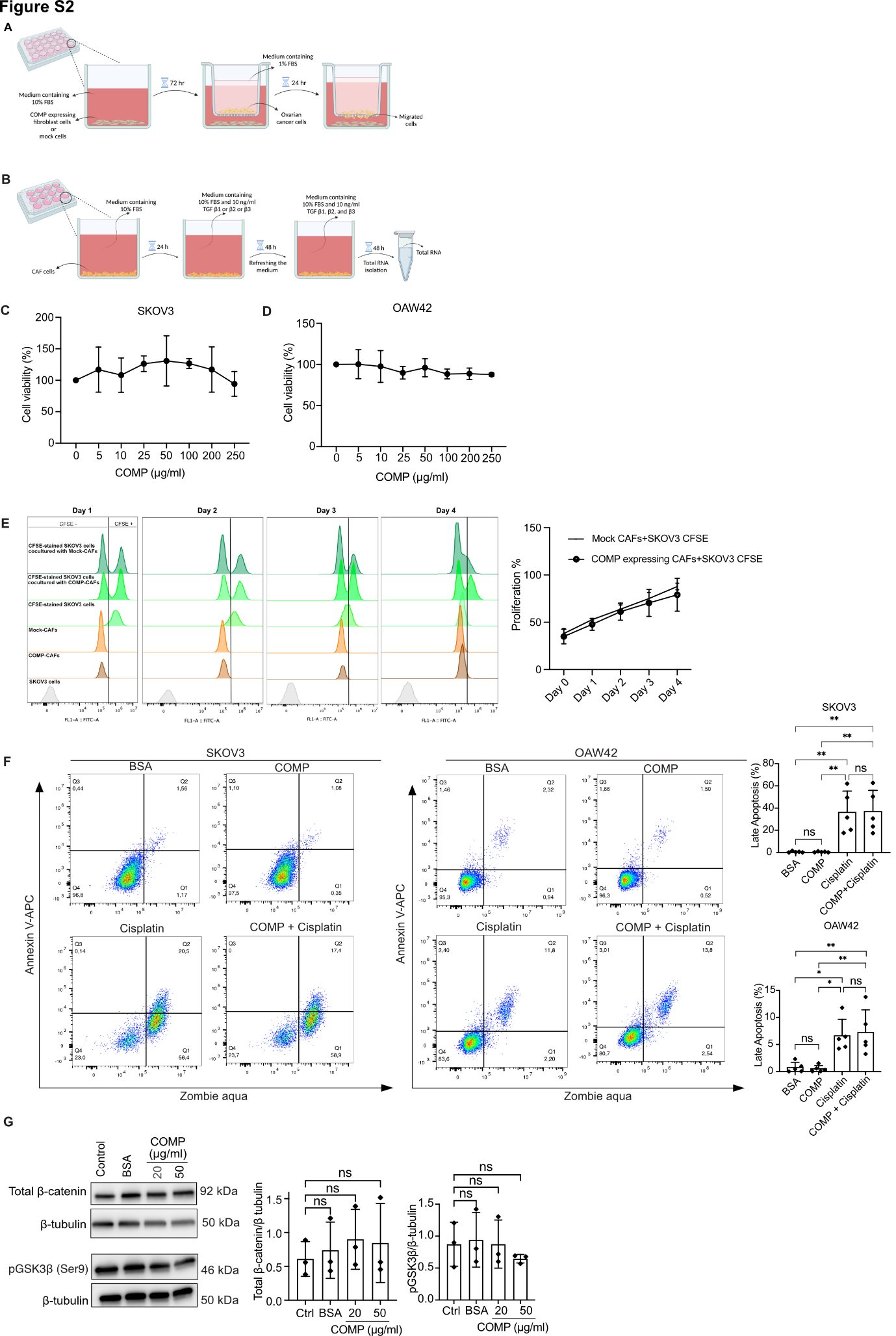
**

**Figure S2.** Graphical schemes representing the co-culture assay of COMP-expressing CAFs with ovarian cancer cell lines (**A**) and CAFs treatment with TGF-β isoforms (**B)**. **C, D.** Cell proliferation assay for SKOV3 and OAW42 cells treated with increasing concentrations of COMP using CyQUANT cell proliferation assay kit. Data were normalized to PBS-treated cells. The *p*-value was calculated by one-way ANOVA followed by Dunnett’s post-test (n=3, mean±SD). **E.** Cell proliferation assay for CFSE-labeled SKOV3 cells cocultured with COMP-expressing CAFs or the mock control CAFs using flowcytometry. The *p*-value was calculated by two-way ANOVA followed by Sidak’s post-test (n=3, mean±SD). **F.** Apoptosis assay using Annexin V-APC and Zombie aqua dye in four samples, including BSA (20 μg/ml)-treated, COMP (20 μg/ml)-treated, cisplatin-treated, and COMP in combination with cisplatin-treated (COMP+cisplatin) of SKOV3 and OAW42 cells. SKOV3 and OAW42 cells were treated with 10 μM and 20 μM of cisplatin, respectively. The *p*-value was calculated by one-way ANOVA followed by Sidak’s post-test (n=3, mean±SD). **G.** Evaluation of total β-catenin and pGSK3β protein expression levels in SKOV3 cells treated with COMP (20 and 50 μg/ml) or BSA (50 μg/ml) as a control by western blotting. PBS-treated cells were also used as a control. β-tubulin served as a loading control. The *p*-value was calculated by one-way ANOVA followed by Dunnett’s post-test. The data are representative of three independent experiments and graphs depict mean±SD. (*, **, and ns indicate *p*<0.05, *p*<0.01, and non-significant, respectively)

Table S1. List of the used antibodies

| Antibody | Company | Cat number | Clone | Method |
| --- | --- | --- | --- | --- |
| Anti-human NOTCH1 | Cell Signaling Technology | 3608 | D1E11 | WB |
| Anti-human NOTCH2 | Cell Signaling Technology | 5732 | D76A6 | WB |
| Anti-human NOTCH3 | Cell Signaling Technology | 5276 | D11B8 | WB |
| Anti-GAPDH | Abcam | Ab8245 | 6C5 | WB |
| Anti-human JAGGED 1 | R&D Systems | AF1277 | Polyclonal | PLA |
| Anti-human NOTCH3 ECD | Novus Biologicals | H00004854-M01 | 1G5 | PLA |
| Anti-active-β-catenin | Cell Signaling Technology | 8814 | D13A1 | WB |
| Anti-pGSK3β (Ser9) | Cell Signaling Technology | 5558 | D85E12 | WB |
| Anti-β-catenin | BD Bioscience | 610154 | 14/Beta-Cat. | WB |
| Anti- β tubulin | Abcam | Ab6046 | Polyclonal | WB |
| Alexa Fluor 488 goat anti rabbit IgG | Invitrogen | A-11008 | Polyclonal | Flow cytometry |
| Anti-Pan Cytokeratin | Merck | ZMS1063 | AE1 | IHC |
| Anti-CD44 | Abcam | Ab157107 | Polyclonal | IHC |
| Mouse IgG2A Isotype Control | R&D Systems | MAB003 | Mouse IgG2A#20102 | PLA |
| Normal Goat IgG Isotype Control | R&D Systems | AB-108-C | Ployclonal | PLA |
| Anti-mouse immunoglobulins/ HRP | Dako | P0447 | Polyclonal | WB |
| Anti-Rabbit immunoglobulins/ HRP | Dako | P0448 | Polyclonal | WB |

Table S2: Significant deregulated EMT-specific genes in SKOV3 cell line treated with recombinant COMP (20 μg/ml) or BSA (20 μg/ml) obtained by RT-qPCR array

| Gene | P value | Fold change | Mean of COMP | Mean of Mock |
| --- | --- | --- | --- | --- |
| ACTB | **0,04887** | **1,809570739** | **0,224551** | **0,124091** |
| AKT1 | **0,000415** | **3,164660804** | **0,0441648** | **0,0139556** |
| B2M | 0,024026 | 2,456868261 | 1,47194 | 0,599111 |
| BMP1 | 0,000826 | 2,515309049 | 0,0112241 | 0,00446233 |
| BMP2 | **0,018636** | **4,391426025** | **0,00186731** | **0,000425217** |
| BMP7 | 0,045438 | 2,691087898 | 0,000015811 | 5,87531E-06 |
| CALD1 | **0,00426** | **3,881231275** | **0,00427624** | **0,00110177** |
| CAMK2N1 | 0,0171 | 3,014871375 | 0,0113147 | 0,00375296 |
| CAV2 | **0,007696** | **2,053039516** | **0,165722** | **0,0807201** |
| CDH2 | 0,000699 | 3,058084213 | 0,0069727 | 0,00228009 |
| COL5A2 | 0,016778 | 2,339250646 | 0,000212745 | 9,09459E-05 |
| CTNNB1 | 0,007685 | 2,439797963 | 0,0217847 | 0,00892891 |
| DSP | 0,006345 | 3,018315593 | 0,0847772 | 0,0280876 |
| EGFR | 0,002552 | 4,872146864 | 0,0316901 | 0,00650435 |
| ESR1 | 0,001317 | 3,26585768 | 0,00178021 | 0,000545099 |
| GNG1 | 0,032368 | 2,571845214 | 0,182122 | 0,0708136 |
| GSC | 0,000703 | 3,696278683 | 0,000325515 | 8,80656E-05 |
| GSK3B | **0,00874** | **3,835283044** | **0,0335282** | **0,00874204** |
| IGFBP4 | **0,001662** | **5,864300125** | **0,00065981** | **0,000112513** |
| IL1RN | **0,006527** | **5,580251116** | **0,000231669** | **4,15159E-05** |
| ILK | **0,001319** | **2,869449837** | **0,0177939** | **0,00620115** |
| ITGA5 | **0,009271** | **2,308141994** | **0,00777471** | **0,00336838** |
| ITGAV | **0,011049** | **4,375298736** | **0,0507596** | **0,0116014** |
| ITGB1 | 0,027858 | 1,832563385 | 0,355067 | 0,193755 |
| JAG1 | 0,012583 | 3,606485608 | 0,0266836 | 0,00739877 |
| KRT14 | 0,008248 | 0,519010125 | 0,00271322 | 0,00522769 |
| KRT7 | **0,034065** | **4,084346955** | **0,344976** | **0,0844629** |
| MAP1B | 0,035301 | 2,921645549 | 0,00961375 | 0,00329053 |
| MMP2 | **0,012389** | **3,164511435** | **0,00814136** | **0,00257271** |
| NUDT13 | 0,017465 | 6,261884825 | 0,00107936 | 0,00017237 |
| OCLN | **0,002822** | **6,314572817** | **0,0324204** | **0,00513422** |
| PDGFRB | 0,013768 | 1,873805542 | 3,28653E-06 | 1,75393E-06 |
| PLEK2 | **0,041367** | **3,761757579** | **0,0137589** | **0,00365757** |
| PPPDE2 | 0,005272 | 3,699073495 | 0,00416099 | 0,00112487 |
| PTK2 | 0,007964 | 2,958411085 | 0,0189429 | 0,00640305 |
| PTP4A1 | 0,002412 | 3,57479473 | 0,00711507 | 0,00199034 |
| RAC1 | 0,001858 | 2,038138353 | 0,134958 | 0,0662161 |
| RGS2 | 0,029706 | 2,753754911 | 0,000310254 | 0,000112666 |
| RPLP0 | 0,029476 | 1,720000904 | 0,411322 | 0,239141 |
| SMAD2 | 0,000274 | 3,08057595 | 0,0503204 | 0,0163347 |
| SNAI2 | **0,033136** | **1,859049605** | **0,00227255** | **0,00122243** |
| SNAI3 | 0,003744 | 2,870524811 | 5,08147E-05 | 1,77022E-05 |
| SPP1 | 0,035881 | 0,571153065 | 0,045152 | 0,0790541 |
| STAT3 | 0,006928 | 1,824323808 | 0,0894491 | 0,0490314 |
| STEAP1 | **0,008521** | **7,525174454** | **0,00252524** | **0,000335572** |
| TBP | 0,02274 | 5,344994046 | 0,0138124 | 0,00258417 |
| TCF4 | 0,025991 | 1,872388858 | 1,11619E-05 | 5,96131E-06 |
| TMEFF1 | 0,034375 | 2,075038786 | 0,0139475 | 0,00672156 |
| WNT1 | 0,008671 | 3,622664338 | 7,42552E-05 | 2,04974E-05 |
| ZEB1 | 0,027975 | 3,384244056 | 0,0252328 | 0,00745597 |

Bold values represent statistically significant genes in both cell lines, SKOV3 and OAW42.

Table S3: Significant deregulated EMT-specific genes in OAW42 cell line treated with recombinant COMP (20 μg/ml) or BSA (20 μg/ml) obtained by RT-qPCR array

| Gene | P value | Fold change | Mean of COMP | Mean of Mock |
| --- | --- | --- | --- | --- |
| ACTB | **0,001279** | **2,088847** | **0,528309** | **0,252919** |
| AKT1 | **0,008556** | **1,441282** | **0,039448** | **0,02737** |
| BMP2 | **0,023011** | **2,123421** | **0,000915** | **0,000431** |
| CALD1 | **0,021975** | **1,792572** | **0,00443** | **0,002471** |
| CAV2 | **0,023099** | **1,257134** | **0,044894** | **0,035712** |
| CDH1 | 0,022346 | 0,397049 | 7,56E-05 | 0,00019 |
| COL1A2 | 0,030191 | 2,605856 | 0,000387 | 0,000149 |
| GSK3B | **0,013436** | **1,327286** | **0,011817** | **0,008903** |
| IGFBP4 | **0,043881** | **0,540391** | **1,00E-05** | **1,86E-05** |
| IL1RN | **0,0064** | **0,561683** | **1,35E-05** | **2,40E-05** |
| ILK | **0,013035** | **1,273912** | **0,010252** | **0,008048** |
| ITGA5 | **0,007641** | **2,355965** | **0,005526** | **0,002345** |
| ITGAV | **0,0195** | **1,537005** | **0,022501** | **0,014639** |
| KRT7 | **0,000831** | **1,635563** | **0,400327** | **0,244764** |
| MMP2 | **0,046818** | **1,941186** | **0,00145** | **0,000747** |
| MMP9 | 0,045787 | 0,536879 | 7,10E-07 | 1,32E-06 |
| MSN | 0,0161 | 1,232828 | 0,109877 | 0,089126 |
| OCLN | **0,022383** | **0,640229** | **0,003031** | **0,004734** |
| PLEK2 | **0,001306** | **1,57184** | **0,006541** | **0,004162** |
| SERPINE1 | 0,000371 | 4,595882 | 0,049558 | 0,010783 |
| SNAI2 | **0,007321** | **3,203151** | **0,005819** | **0,001817** |
| STEAP1 | **0,03011** | **1,264263** | **0,000584** | **0,000462** |
| TFPI2 | 0,007828 | 1,471057 | 0,039211 | 0,026655 |
| TGFB2 | 0,01334 | 2,057896 | 0,000889 | 0,000432 |
| TSPAN13 | 0,009343 | 1,333673 | 0,002737 | 0,002052 |
| VIM | 0,000036 | 1,755155 | 0,527379 | 0,300474 |
| ZEB2 | 0,038989 | 2,093227 | 3,85E-05 | 1,84E-05 |

Bold values represent statistically significant genes in both cell lines, SKOV3 and OAW42.
